# Blood Donation Screening and West Nile Virus Surveillance Strategy in France

**DOI:** 10.1001/jamanetworkopen.2025.24494

**Published:** 2025-07-31

**Authors:** Gilda Grard, Florian Franke, Syria Laperche, Amandine Cochet, Marie-Claire Paty, Gaëlle Gonzalez, Fanny Chaubenit, Camille Victoire Migné, Albin Fontaine, Sophie Le Cam, Xavier De Lamballerie, Pierre Gallian, Guillaume André Durand

**Affiliations:** 1 Etablissement Français du Sang, Saint-Denis, France; 2 Unité des Virus Émergents, Aix-Marseille University, IRD 190, Inserm 1207, Marseille, France; 3 French Armed Forces Biomedical Research Institute, Valérie-André, Brétigny-sur-Orge, France; 4 Santé Publique France, Saint-Maurice, France; 5 ANSES, INRAE, Ecole Nationale Vétérinaire d’Alfort, UMR 1161 Virologie, Laboratoire de Santé Animale, Maison-Alfort, France; 6 Agence régionale de santé Provence-Alpes-Côte d’Azur, Délégation départementale du Var, Toulon, France

## Abstract

This case series describes the use of nucleic acid testing in screening blood donations for West Nile virus in areas of France in which the virus circulated in previous years.

## Introduction

West Nile virus (WNV) is an orthoflavivirus transmitted by *Culex* species mosquitoes maintaining an enzootic cycle in birds. Humans and equids are incidental hosts and epidemiologic dead ends. In Europe, WNV circulation has been expanding annually, with persistent endemic activity and periodic epidemics, frequently affecting new areas.^1^

West Nile virus infection is mainly asymptomatic (80%) in humans and equids, but severe neurologic manifestations have been reported in vulnerable patients. French surveillance has included detection of WNV in humans; equines; birds; and opportunistically, mosquitoes. Human surveillance is based on mandatory notification of the infection, which is enhanced from May 1 to November 30 in high-risk areas by health professional awareness and monitoring by the National Reference Centre for Arboviruses. Animal health surveillance primarily targets the detection of syndromic cases in horses and birds, while entomologic surveillance remains sporadic and experimental.

Transmission by substances of human origin, especially after blood transfusion, has been reported.^2^ According to national recommendations, blood safety measures rely on individual screening of blood donations, introduced after the first report of an autochthonous human case in the affected department.^3^

## Methods

This case series was performed according to French regulation. Ethical review and approval were not required in accordance with local legislation and institutional requirements. The study followed the reporting guideline for case series. To ensure blood supply self-sufficiency and availability during the period of the Olympic Games, the French public transfusion service introduced WNV nucleic acid testing from July 1 to September 15, 2024, for blood donations collected in areas where the virus circulated in the previous years. Four departments in the South of France were included: Bouches-du-Rhône, Gard, Hérault, and Var. Notably, Bouches-du-Rhône and Gard include the Camargue region, a vast wetland with an important staging point for migratory birds.^4^

## Results

From all departments combined, there were approximately 56 000 blood donations. In the Var department, the first human WNV case was detected on week 29 through blood donation testing, 2 weeks before the first detection of WNV in equine and mosquito excreta^5^ (Figure) and 3 weeks before the first human symptomatic case. In this department, a total of 24 human cases were reported, including 23 in a 100-km^2^ area (10 people with neurologic disease, 9 with WNV fever, 5 with asymptomatic infection). In Camargue, the first signal indicating WNV circulation was also observed among blood donors on week 34 in the Gard department, 2 weeks before the detection of the first symptomatic human case. A total of 10 symptomatic human cases (9 people with WNV fever, 1 person with neurologic disease) and 73 equine cases were reported. The virus was also detected in mosquito traps.

**Figure.**
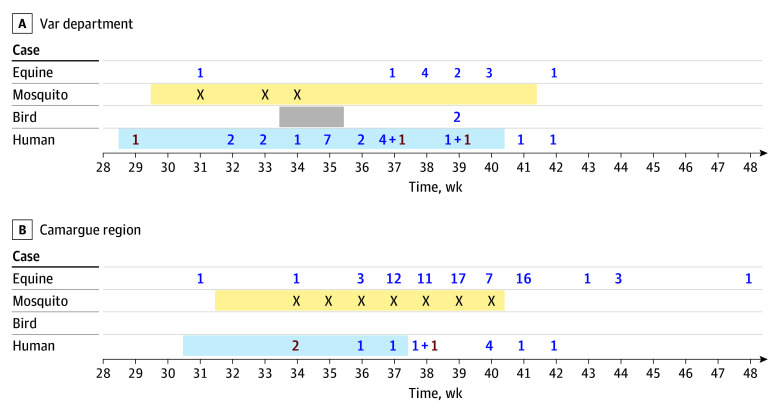
Number of Alerts for West Nile Virus Detection in Humans, Birds, Equines, and Mosquitoes Red numbers indicate blood donors, and blue numbers indicate symptomatic cases. The *X*s indicate virus detection in mosquito traps. Blue shading indicates the period from the date of blood donation or the date of symptom onset from the first case to the date of symptom onset of the last case. Yellow shading indicates the period from the deposit time of the first mosquito trap to the date of the last deposit. Gray shading indicates the period in which bird mortality was observed.

## Discussion

Given the rising number of human WNV cases in the context of global warming, coupled with the high proportion of asymptomatic forms,^6^ this case series questioned the relevance of current human surveillance, which primarily focuses on symptomatic cases. The preventive blood donation nucleic acid testing strategy used during the Olympic Games allowed active surveillance for early WNV detection in humans. In areas with the highest number of human cases, the first WNV detection was through blood donation screening, occurring before the detection of symptomatic cases. Notably, in the Var department, detection in the blood donor with infection was 2 weeks before the appearance of equine, bird, and mosquito cases.

A limitation of this study was that most blood donors live in urban areas, which may not represent the most exposed population. To improve our national WNV human surveillance system and enable early preventive measures to avoid transmissions by substances of human origin, a strategy that combines systematic WNV screening in symptomatic cases with nucleic acid testing in blood donors in high-risk areas may be relevant. The place, cost, and benefits of implementing WNV nucleic acid screening of blood donations integrated in One Health surveillance should be assessed.

## Notes

Supplement.
Data Sharing Statement
