# Supplementary material for: Blood Donation Screening and West Nile Virus Surveillance Strategy in France
Source: JAMA Netw Open. 2025 Jul 31;8(7):e2524494. doi: 10.1001/jamanetworkopen.2025.24494 (PMC12314714; doi:10.1001/jamanetworkopen.2025.24494)
Supplement: Supplement. — Data Sharing Statement [file jamanetwopen-e2524494-s001.pdf]

Data Sharing Statement

Grard. Improvement of the West Nile Virus Surveillance Strategy in France. *JAMA Netw Open*. Published July 31, 2025. doi:10.1001/jamanetworkopen.2025.24494

Data
Data available: No
